# Quantitative metabolomics of the thermophilic methylotroph *Bacillus methanolicus*

**DOI:** 10.1186/s12934-016-0483-x

**Published:** 2016-06-01

**Authors:** Marc Carnicer, Gilles Vieira, Trygve Brautaset, Jean-Charles Portais, Stephanie Heux

**Affiliations:** Université de Toulouse; INSA, UPS, INP; LISBP, 135 Avenue de Rangueil, 31077 Toulouse, France; INRA, UMR792, Ingénierie des Systèmes Biologiques et des Procédés, 31400 Toulouse, France; CNRS, UMR5504, 31400 Toulouse, France; Department of Biotechnology, Norwegian University of Science and Technology, Trondheim, Norway; LISBP–INSA de Toulouse, 135 Avenue de Rangueil, 31077 Toulouse Cedex 04, France

**Keywords:** *Bacillus methanolicus*, Methanol, Quantitative metabolomics, Quenching

## Abstract

**Background:**

The gram-positive bacterium *Bacillus methanolicus* MGA3 is a promising candidate for methanol-based biotechnologies. Accurate determination of intracellular metabolites is crucial for engineering this bacteria into an efficient microbial cell factory. Due to the diversity of chemical and cell properties, an experimental protocol validated on *B. methanolicus* is needed. Here a systematic evaluation of different techniques for establishing a reliable basis for metabolome investigations is presented.

**Results:**

Metabolome analysis was focused on metabolites closely linked with *B. methanolicus* central methanol metabolism. As an alternative to cold solvent based procedures, a solvent-free quenching strategy using stainless steel beads cooled to −20 °C was assessed. The precision, the consistency of the measurements, and the extent of metabolite leakage from quenched cells were evaluated in procedures with and without cell separation. The most accurate and reliable performance was provided by the method without cell separation, as significant metabolite leakage occurred in the procedures based on fast filtration. As a biological test case, the best protocol was used to assess the metabolome of *B. methanolicus* grown in chemostat on methanol at two different growth rates and its validity was demonstrated.

**Conclusion:**

The presented protocol is a first and helpful step towards developing reliable metabolomics data for thermophilic methylotroph *B. methanolicus.* This will definitely help for designing an efficient methylotrophic cell factory.

**Electronic supplementary material:**

The online version of this article (doi:10.1186/s12934-016-0483-x) contains supplementary material, which is available to authorized users.

## Background

Industrial biotechnology mainly uses sugars and molasses as carbon sources. These raw materials come from plants and require cultivable land which is increasingly needed to produce food for human populations. The possibility of using non-food raw materials, such as one-carbon (C1) substrates, as alternative feedstock in microbial fermentation for the manufacturing of special, fine, bulk, and fuel chemicals has attracted considerable biotechnological and scientific interest. C1 compounds such as methane and methanol occur abundantly throughout nature, and in contrast to molasses, methanol is a pure raw material which can be completely consumed by methylotrophic bacteria during fermentation [1].

Methylotrophic bacteria have already been identified as potential producers for biotechnological processes [1]. Among the different microorganisms able to use C1 sources, the gram-positive facultative methylotroph *Bacillus methanolicus* is a possible cell factory for the industrial production of l-lysine, l-glutamate and cadaverine from methanol at elevated temperatures [2–4]. *B. methanolicus* MGA3 (ATCC 53907) growths at 50 °C and assimilates methanol by using the ribulose monophosphate (RuMP) pathway [5]. Genes involved in this metabolic pathway are located in the pBM19 plasmid and are upregulated upon growth on methanol compared to mannitol [6]. Recently, different isoenzymes present in the RuMP pathway have been biochemically characterized providing evidence for the importance of plasmidic isoenzymes in methanol-based growth [7–9]. The *B. methanolicus* genome sequence is now complete and provides physiological and metabolic traits that pave the way for system-level metabolic engineering [6]. Both proteome and transcriptome analyses have recently been used to analyse global gene regulation upon methylotrophic growth in this organism [10, 11]. However, there are still gaps in our biochemical and regulatory understanding of how *B. methanolicus* efficiently uses methanol as sole source of carbon and energy.

Metabolomics is defined as the comprehensive analysis of the metabolites produced by an organism. Because the metabolome is the consequence of the amplification and integration of the other ‘omic’ levels, it provides information on cellular activity and instantaneous snapshots of cell physiology [12]. In addition, as metabolites are the functional entities within cells, genetic modifications or changes in the cell’s environment have a direct influence on their levels. Therefore, assessing changes in metabolite levels in wild type vs. engineered strains or under different culture conditions may help elucidate limiting or inhibiting biosynthetic steps as well as advance our understanding of cellular metabolism [13]. This explain why metabolomics has become a major tool in metabolic engineering for strain improvement.

All procedures used for metabolome analysis have the same operational sequence, i.e. culture broth is sampled, cell metabolism is quenched and the metabolites are extracted from the cells. Because of the rapid biological turnover of the metabolites (from subseconds to 100 s), the sampling and quenching steps have to be fast to properly stop the metabolic activity of the organism. Rapid sampling of the culture broth in a bioreactor can be achieved manually or automatically with sampling devices and stopped flow sampling systems [14–16]. Several quenching procedures exists which can be divided into two main groups: with or without cell separation. In the first group, the sample is mixed with cold solvent and the cells are separated from the culture medium by centrifugation or filtration [17, 18]. The cells are then washed and resuspended in extraction solution (i.e. cold or hot solvent) [14, 15]. The main limitation of such approaches is the leakage of intracellular metabolites into the solution due to damage to the membrane and cell walls when the cells are in contact with the quenching solution [19]. The extent of leakage is determined by different factors including time of exposure, quenching temperature, the properties of the cold aqueous methanol solution (e.g. ionic strength, concentration of methanol; [17]) and the physical–chemical properties of the metabolites (e.g. size and polarity; [20]). To prevent leakage, fast filtration methods collect cells before quenching [16]. In this case, the cells are first separated from the culture medium by vacuum filtration, the filter is then washed using an appropriate solution to get rid of extracellular medium, and the cells are then transferred into cold organic solvent to quench the metabolism [16, 21]. However, these methods do not allow immediate quenching of metabolic activity [19, 21, 22]. In the second group, the cell separation step is skipped, and quenching and extraction are performed simultaneously using appropriate buffers [15, 21]. This can even be extended to a fully integrated approach which allows quenching and extraction during sampling [23]. In contrast to cell separation methods, which give direct access to the levels of intracellular metabolites, here, the levels of extracellular metabolites have to be quantified [15, 21]. In these approaches, the levels of metabolites in the cells are estimated by the so-called ‘‘differential method’’ by subtracting the amount in the extracellular medium from the total amount in the whole broth. Using such integrated procedures circumvents leakage phenomena while allowing a sub-second arrest of metabolic activity; however, the precision of the measurements of intracellular metabolites is significantly reduced [19, 21].

Unfortunately, due to the vast diversity of chemical and cell properties, no universal method exists for metabolome analysis of bacterial cells, and protocols have to be adapted and evaluated for each individual organism [17, 18, 21, 24]. In this study, we established the first method for proper metabolome quantification in *B. methanolicus*. A solvent-free quenching strategy using stainless steel beads cooled to −20 °C was assessed as a possible alternative to cold solvent based approaches [25]. The cold stainless steel bead sampling method has been proven to be suitable when rapid sampling and arrest of cells are required when following the dynamics of substrate uptake rate in the short sampling time frame [25, 26]. Cold beads can thus be a good strategy for efficient metabolic arrest for quantifying intracellular metabolites pools. The applicability of protocols with and without cell separation was evaluated with respect to their precision, the consistency of the measurements, and the extent of leakage of metabolites from quenched cells. To this end, a quantitative mass balance approach was used to monitor the fate of metabolites during processing of the samples [17, 18]. The best protocol was then used to differentiate the relevant metabolome of *B. methanolicus* MGA growing on methanol at two different growth rates, thereby providing new valuable insights into the methylotrophic properties of this bacterium.

## Methods

### Strain and culture conditions

#### All analytical grade reagents were supplied by Sigma-Aldrich

In this study, the gram-positive methylotrophic bacterium *B. methanolicus* wild-type MGA3 (ATCC 53907) strain was used. Chemostat cultures were performed in 0.5 litre bioreactors (*INFORS HT Multifors,* The Netherlands) with a working volume of 0.4 litres, coupled to a Dycor ProLine Process Mass Spectrometer (AMETEK Process Instruments, USA). The culture medium was derived from [27]. The medium per litre in shake flask pre-cultures and in the batch phase of the cultures was: 8.42 g Na_2_HPO_4_·12 H_2_0, 1.47 g KH_2_PO_4_, 2.11 g (NH_4_)_2_SO_4_, 0.25 g yeast extract, 1 ml of 1 M MgSO_4_ solution, 1 ml of trace salt solution, 1 ml of vitamin solution, 0.05 ml Antifoam 204 (Sigma-Aldrich) and 120 mM of methanol. The trace salt solution per litre was: 5.56 g FeSO_4_·7 H_2_O, 0.027 g CuCl_2_·2 H_2_O, 7.35 CaCl_2_·2 H_2_O, 0.040 g CoCl_2_·6 H_2_O, 9.90 g MnCl_2_·4 H_2_O, 0.288 g ZnSO_4_·7 H_2_O and 0.031 g H_3_BO_3_. The vitamin solution per litre was: 0.10 g d-biotin, 0.10 g thiamine·HCl, 0.10 g riboflavin, 0.10 g pyridoxine·HCl, 0.10 g pantothenate, 0.10 g nicotinamide, 0.02 g p-aminobenzoic acid, 0.01 g folic acid, 0.01 g vitamin B12 and 0.01 g lipoic acid. The culture medium used in the continuous phase was the same as in the batch phase but without the yeast extract.

First, half litre shake flasks containing 150 ml of the pre-culture medium were inoculated with cryostock of *B. methanolicus* cells. The cultures were grown overnight at 50 °C under shaking at 200 rpm, and used to inoculate the reactors. After complete termination of the batch phase, approximately 7 h after inoculation, the chemostat phase was started. In this phase, the cells were grown under carbon-limited conditions at specific dilution rates (D), 0.10 h^−1^ or 0.15 h^−1^. The aeration rate of 1 vvm was controlled by a mass flow meter (*INFORS HT Multifors*, The Netherlands) and pO_2_ was maintained above 25 % throughout culture. Temperature, pH and stirring speed were maintained at 50 °C, pH 6.5 (with KOH 1 M) and 800 rpm, respectively. The N_2_, O_2_, Argon, CO_2_ and methanol concentrations in the bioreactor off-gas were measured on-line with the mass spectrometer.

### Sampling, quenching and extraction

**Fig. 1:**
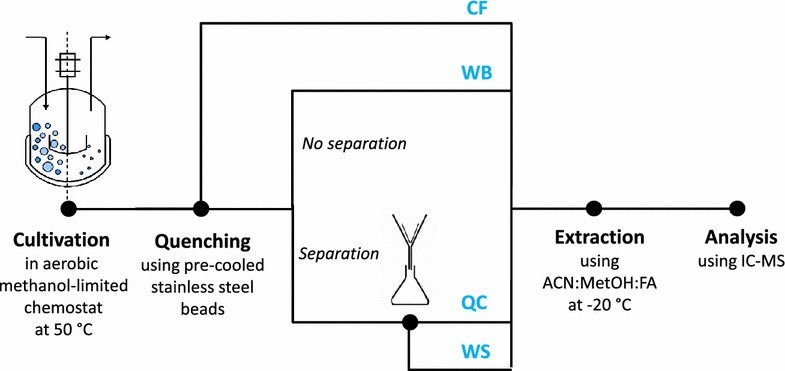
Overview of the methods tested for metabolome samples in *B. methanolicus* (see Sect. ''Methods'' for details). *CF* quenched culture filtrate sample; *WB* quenched whole broth sample; *QC* quenched and washed cells separated from the medium by filtration; *WS* washing solution sample; *CAN* acetonitrile, *MetOH* methanol; *FA* formic acid, *IC*-*MS* Ionic chromatography-mass spectrometry

Figure 1 shows the samples taken to evaluate the methods for metabolome quantification in *B. methanolicus.* Whatever the type of sample, quenching was done using seven pre-cooled (−20 °C if not stated differently) stainless steel beads (4 mm diameter, Saluc, Belgium). The mass of beads used per mass of liquid ranged between 4 and 10 g/g depending on the volume sampled from the culture. All the metabolites were extracted using 3 ml of a solution containing acetonitrile : methanol : 0.1 M formic acid, 40:40:20 v/v and left at −20 °C for 1 h. All the extracted samples were stored at −80 °C until further treatment.

*Quenched whole broth (WB) samples* Samples were taken directly from the bioreactor using a 1 ml syringe containing seven pre-cooled (−20 °C) stainless steel beads. The samples were then placed in a tube containing the extracting solution and vortexed to homogenise the sample. The exact volumes sampled were determined by weighing the extracting tubes before and after sampling. U-^13^C-labeled *Escherichia coli* cell extract (200 µl) was added to the extracting tube as internal standard. Three technical replicates per culture were performed. The average time between sample collection and extraction was 21 ± 2 s.

*Quenched culture filtrate (CF) samples* Culture filtrate samples were obtained using a 10 ml syringe attached to a 0.20 µm filter (Minisart, Sartorius). The samples were directly filtered during their removal from the bioreactor sampling port. Seven pre-cooled (−20 °C) stainless steel beads were placed inside the syringe. Next, the filtrate solution was moved to an ice-cooled Eppendorf tube from which 200 µl was dispensed into an extracting tube together with 200 µl of U-^13^C-labeled *E. coli* cell extract. Three technical replicates per culture were performed. The average time between sample collection and extraction was 46 ± 1 s.

*Quenched and washed cells separated from the medium by filtration (QC) and washing solution (WS) samples* These samples were taken in the same way as the WB samples and transferred to ice-cooled Eppendorf tubes using syringes containing seven stainless steel beads precooled to −20 °C (QC-20) or to 4 °C (QC-4). From the Eppendorf tubes, 200 µl of culture were transferred to membrane disk filters (0.2 µm pore size, Sartolon Polyamid, Sartorius) and filtered using a vacuum pump. The filters were washed twice with 1 ml of cooled (4 °C) medium without vitamins, trace salts or methanol. After washing, the filters were rapidly transferred to extracting tubes together with 200 µl of U-^13^C-labeled *E. coli* cell extract. The washing solution for the two quenching temperatures (4 °C and −20 °C) was collected and mixed with 200 µl of U-^13^C-labeled *E. coli* cell extract (WS-4 and WS-20). Duplicate technical samples were taken in both protocols tested (QC-20 + WS-20 and QC-4 + WS-4). The average time between sample collection and extraction was 105 ± 2 s.

### Measurements of intracellular metabolite levels

Extracted samples were evaporated in a Rotavapor (Büchi, Switzerland) for approximately 15 h until complete dryness. The samples were then re-dissolved in 400 µl of ultrapure water and stored at −20 °C until analysis. Further dilutions were performed, if needed, to adjust the salt content of the samples to avoid ion suppression during MS analysis.

*Metabolome analysis* Intracellular metabolites content of the WB, CF, QC and WS samples were analyzed as previously described [21, 28]. Briefly a Dionex ICS 2000 system (Dionex, Sunnyvale, USA) coupled to a triple quadrupole QTrap 4000 mass spectrometer (Applied Biosystems, Foster City, USA) was used. Peak integration was performed manually using Analyst 1.5.2 software (Sierra Analytics, USA). All samples were analyzed in the negative mode by multiple reactions monitoring (MRM). To ensure highly accurate quantification, the isotope dilution mass spectrometry (IDMS) method was used [29]. Integration of all the peaks were performed manually using the Analyst 1.5.2 software (Sierra Analytics, USA). The number of samples collected and analysed for each experiment is given in Additional file 1.

*Metabolome quantification* The quantification of intracellular metabolites in all biological samples was done using a program developed in R [30]. Briefly, a calibration curves (^12^C analyte peak area/^13^C analyte peak area) were performed using 8 concentrations ranging from 0.04 μM until 10.0 μM. The concentration (Ce) of each analyte in (μmol/L) in the extract was determined using the following equation: Ce = (y − b)/a with y = ^12^C peak area/^13^C peak area. The calibration coefficients [i.e. the slope (a), the intercept (b) and the correlation coefficient (r^2^)] for each analyte are given in Additional file 1. The metabolite concentrations in μmol/gDCW for each replicate were calculated using the dry cell weight measurement and the weighted sample volume taken from the bioreactor as described in Additional file 1. The mean of concentrations of each metabolite and the standard error were calculated for each sample type. For the differential method, the intracellular concentration was obtained by the difference between the WB and CF and the error was obtained by propagation on the standard error.

Our analysis of the metabolome of *B. methanolicus* focused on metabolites closely related with the central methanol metabolism. The complete list is given in Additional file 1.

### Measurements of extracellular metabolite levels

Initial culture media and culture samples collected at different times were filtered (Minisart 0.2 µM filter from Sartorius, Göttingen, Germany). Supernatant fraction were prepared for 1D 1H NMR analysis, by adding 100 μL of Deuterated trimethylsilyl propionate (TSPd4) at 4,3 mM diluted in DH_2_O to 500 μL of supernatant. 1D 1H NMR spectra were acquired on a Bruker Ascend 800 MHz magnet (Bruker, Germany) using a 5 mm CPQCI cryoprobe 1H-31/13C/15 N/Z GRD. A sequence using presaturation (ZGPR) was used for water signal suppression, with a 30° pulse angle and a relaxation delay between scans of 10 s to ensure full signal recovery. A total of 32 scans were accumulated (after 4 dummy scans) with 292 K data points, 6.83 s of acquisition time, 5 s of recycle delay and no spin. Using Topspin 2.1 (Bruker, Rheinstatten, Germany), the FIDs were zero-filled, Fourier transformed with 0.5-Hz exponential line broadening, manually phase corrected, automatically baseline corrected, and aligned to the TSPd4 signal. Topspin 2.1 (Bruker, Rheinstatten, Germany) was also used for peak integration. Metabolite quantification was performed using a program developed in R [30]. Three samples were collected and analyzed for each dilution rate.

### Dry cell weight (DCW) analysis

Samples (3 × 5 ml) of culture broth were taken from the bioreactors and filtered with a pre-weighted membrane disk filter (0.2 µm pore size, Sartolon Polyamid, Sartorius) using a vacuum pump. No washing steps were performed to remove salts from the biomass because the chemostat medium contains 11.9 g/l of salt (see above) which is close to isotonic (0.9 %). The membrane disk filters were dried at 80 °C until stable weight.

### Data consistency

*Chemostat cultures* Under the culture conditions applied in this study, *B. methanolicus* biomass and carbon dioxide were the only compounds produced. The consistency of the experimental data was checked using standard data reconciliation procedures under the elemental mass balance constraint [31, 32]. For all chemostat cultures, consistency was acceptable at a confidence level of 95 %, showing there were no gross measurement errors. The biomass elemental composition used in the reconciliation procedure was taken from the closely related non-methylotrophic bacterium *Bacillus subtilis,* CH_1.646_N_0.219_O_0.410_S_0.005_ [33]. Ash contents were considered to be 6 % of the dry cell weight, which is the average value obtained from different microorganisms (i.e. *Escherichia coli, Aspergillus niger, Penicicillium chrysogenum, Klebsiella aerogenes*) [34].

*Consistency of metabolite measurements* The consistency of the metabolome data obtained was checked under the constraint of Eq. 1, as described in [18]. Briefly, the amount of each metabolite was quantified in the different samples (WB, QC, CF and WS). Accordingly, mass balance should be satisfied for each metabolite, *i*, independently of the sampling method used:

1 $${\text{M}}_{\text{i}} \left( {\text{WB}} \right) = {\text{M}}_{\text{i}} \left( {\text{QC}} \right) + {\text{M}}_{\text{i}} \left( {\text{WS}} \right) \,$$

This balance states that the total amount of each metabolite (i.e. the extracellular + intracellular amount) present in WB samples should be equal to the sum of QC and WS measurements. The redundancy of the data set makes it possible to check the statistical consistency using the calculated χ^2^-distributed consistency index *h* [31]. In our conditions, the threshold value of the *h* index with 95 % of confidence level is 9.49. An *h* index above this value is proof of a gross measurement error. All the calculations were performed using R [30].

## Results and discussion

### Cell quenching by solvent free method

Traditionally, cellular metabolism is quenched using cold methanol solvent [17, 21]. However, quenching a methylotrophic metabolism using methanol can affect the results due to its potential assimilation. In addition, this method is clearly not appropriate for gram positive bacteria because it causes intracellular metabolites to leak from the cells into the quenching solution [21]. As an alternative, we evaluated the capacity of pre-cooled steel beads [25] to quench *B. methanolicus* metabolism. In this method, metabolism is supposed to be quenched by exposing the culture broth to stainless steel beads (4-mm diameter) in a syringe precooled to −20 °C. In our conditions, this should induce a sudden drop in temperature of approximately 40–50 °C, which has been reported to halt metabolism in other organisms [15, 17, 18].

To evaluate the feasibility of this quenching procedure, whole broth samples (WB) from a *B. methanolicus* chemostat culture growing at D: 0.10 h^−1^ were taken in triplicate using a 1 ml syringe containing pre-cooled (−20 °C) stainless steel beads (Fig. 1). Considering that metabolite turnover times ranged from a sub-second to seconds and that 21 s were required to process the samples (i.e. from sampling to the extraction step), if enzymatic reactions were still taking place (even at very low rates), the reproducibility of WB samples would be affected. The size of the metabolite pool and the standard error (SE) measured in WB samples are listed in Table 1. As expected, differences between the intracellular metabolite pools were observed as a result of the *B. methanolicus* metabolism. As previously reported for *Corynebacterium glutamicum*, *Saccharomyces cerevisiae* and *Pichia pastoris*, reproducibility was metabolite dependent [18]. 23PG and AMP showed the highest relative standard error (RSE) of 19.4 % and 14.6 %, respectively. However, for the 18 metabolites analysed, the reproducibility of the measurements was on average less than 7 %, indicating no significant variation in the size of the metabolite pool. This demonstrates that metabolic activity is efficiently blocked by the cold stainless beads. This method was therefore used to quench *B. methanolicus* cells.

**Table 1:** Metabolome content of WB samples collected from *B. methanolicus MGA3* grown in methanol-limited chemostat at D: 0.10 h^−1^

	µmol/gDCW ± SE	RSE
23PG	6.1 ± 1.2	19.4
6PG	7.5 ± 1.1	14.3
ADP	7.23 ± 0.10	1.4
AMP	11.9 ± 1.7	14.6
ATP	9.6 ± 0.4	4.2
Cit	9.1 ± 0.9	10.4
F1P	0.9 ± 0.1	8.5
F6P	20.1 ± 0.2	1.1
FBP	15.9 ± 1.2	7.8
Fum	13.4 ± 0.1	0.8
G6P	23.0 ± 3.1	13.5
M6P	1.00 ± 0.02	2.3
Mal	8.77 ± 0.13	1.5
PEP	2.61 ± 0.11	4.2
P-Ser	0.31 ± 0.02	7.4
R5P	13.6 ± 0.4	2.8
S7P	12.3 ± 0.7	5.4
Shi3P	0.17 ± 0.001	0.8
Mean		6.7

*se* standard error; *rse* relative standard deviation in  %

The metabolite abbreviations and the raw data are given in Additional file 1

### Separation versus no separation of cells prior extraction

Once quenched with cold stainless beads, *B. methanolicus* cells can be separated from the cultivation medium prior to extraction or directly mixed with the extraction solution along with their culture medium. In terms of absolute quantification, both protocols have advantages and limitations and choosing one or the other will depend on the extent of leakage from the cells. Protocols based on cell separation make it possible to directly estimate the precise intracellular levels of all metabolites, even those that are largely excreted by the cells. However if leakages do occur, the levels of intracellular metabolites will be underestimated because some of the metabolites are lost in the discarded solutions (i.e. the quenching and washing solutions) [17, 21]. Protocols without cell separation can be used even when leakage occurs, since the intracellular and extracellular metabolites are analysed together. In this case, the level of intracellular metabolites is determined indirectly by subtracting the concentration of extracellular metabolites measured in the culture filtrate from the total pool (intra + extra) in the whole broth sample. However this method can only be used with metabolites that occur in reasonable amounts outside the cell [19, 21].

To assess which protocols can be used to elucidate the metabolome of *B. methanolicus*, two independent chemsotat cultures were performed at D: 0.10 h^−1^ and different types of samples i.e. quenched whole broth (WB), culture filtrate (CF), quenched and washed cells separated from the medium by filtration (QC) and quenching and washing solution (WS) were collected when the culture was in steady state (see Fig. 1). Filtration was preferred to separate quenched cells from the cultivation medium because it has been reported to be more efficient in eliminating extracellular metabolites than centrifugation [35]. In addition, filtration is much faster than centrifugation, hence limiting the period during which metabolites can leak from cells by diffusion over the cell membrane [20]. To assess the sensitivity of *B. methanolicus* to cold shock, i.e. the sudden release of metabolites from the cells when the broth is rapidly cooled [16, 36], two quenching temperatures (i.e. −20 °C and 4 °C) were tested.

#### Detection of metabolite leakage out of the cells

**Fig. 2:**
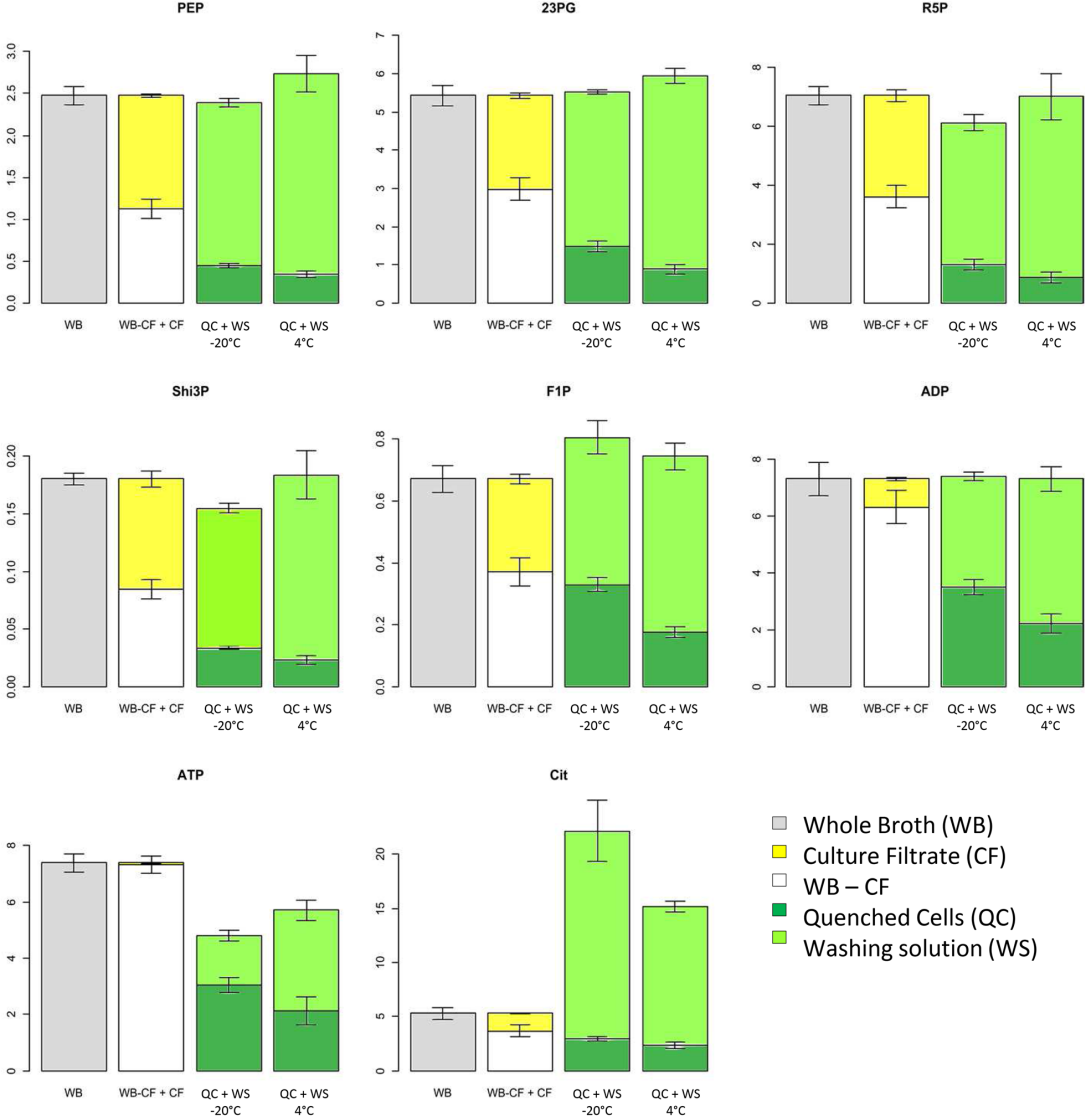
Metabolite balances of representative metabolites from a methanol-limited *B. methanolicus* MGA3 chemostat culture growing at D: 0.10 h^−1^. *Error bars* represent standard errors. The Y axis represents the concentration in µmol/gDCW. The raw data are given in Additional file 1

To check for the occurrence of leakage, quantitative evaluation of the protocols was carried out by mass balance analysis, based on measurements of the metabolites in all the fractions. Figure 2 shows the mass balances for the two protocols and eight selected metabolites with different physical–chemical properties as an illustrative example (see Additional file 1 for complete dataset). Analysis of the different fractions revealed the presence of metabolites both in CF and WS fractions. The presence of metabolites in CF fraction is not surprising and has already been observed for several species [15, 22, 37]. This phenomenon called “extended” overflow metabolism is explained by a passive or active transportation of metabolites from the cells into in the cultivation medium due to a misbalance between carbon uptake and consumption during exponential growth [37]. However, the amount of metabolites in the WS fraction was much higher than in the CF fraction, indicating an additional loss of intracellular metabolites from the cells. This can be explained by the destruction of cell integrity during sample processing. In our experimental setting, cells damages can happen during the quenching step on the freezing stainless steel beads and/or during cell separation by filtration and washing (Fig. 1). Comparison of the extracellular fraction of metabolites obtained with (i.e. WCS-20° and WCS + 4°) or without (WOCS) cells separation indicated a higher fraction of extracellular metabolites for filtrated cells (Table 2). This was amplified when the quenching temperature was set to 4 °C (WCS + 4°). Together this data demonstrated that more intracellular metabolites leaves the cells during the filtration/washing procedures. Extend of leakage depended on the nature of the metabolites but was relatively high for the protocol with cell separation. On average 74 % of the intracellular pool of metabolites were outside the cells against 50 % for the protocol w/o cells separation. This resulted in an underestimation of the intracellular metabolites pools for the filtration based method (QC fraction) compared with the method without cells separation (WB-CF fraction) (Fig. 2).

**Table 2:** Calculated χ^2^-distributed consistency index h, *p* value, extracellular fraction of the different metabolites analysed for the protocols with cells separation with a quenching temperature of −20° (i.e. WCS − 20°); with cells separation with a quenching temperature of 4 °C (i.e. WCS + 4°) and without cells separation with quenching temperature of −20° (WOCS − 20 °C) of the different metabolites analysed

	Extracellular fraction in %			h index	*p* value
	WCS − 20°^a^	WCS + 4°^a^	WOCS − 20°^b^		
23PG	71.5	80.4	47.5	1.71	0789
6PG	85.5	87.6	23.0	1.75	0781
ADP	53.9	64.8	14.0	0.03	1000
AMP	81.9	86.3	91.0	1.52	0823
ATP	31.7	54.5	1.0	13.10	0011
Cit	82.3	83.5	24.0	81.67	<0.0001
F1P	63.4	74.0	52.5	5.13	0275
F6P	80.2	87.2	75.0	0.86	0930
FBP	38.2	61.7	10.0	1.21	0876
Fum	98.7	95.5	99.5	1.27	0866
G6P	91.6	93.5	84.5	0.03	1000
M6P	70.2	79.1	57.0	1.22	0875
Mal	92.1	93.1	89.5	4.96	0292
PEP	81.3	84.4	59.5	0.75	0945
P-Ser	70.0	81.4	17.0	0.10	0999
R5P	82.9	87.2	55.5	0.13	0998
S7P	76.9	87.6	47.5	1.65	0801
Shi3P	76.8	83.7	53.5	0.06	1000

The metabolite abbreviations and the raw data are given in Additional file 1

^a^Ratio between concentrations in the washing solution and in the washing solution plus quenched and washed cells separated from the medium by filtration (WS *100)/(WS + QC)

^b^Ratio between concentrations in the culture filtrate and in the whole broth (CF*100)/WB

We cannot exclude that metabolite leakage occurs also during the quenching procedure as stainless steel beads may cause double stress towards *B. methanolicus*, physical (crash within steel beads) and temperature, which both may affect the cell structure. This should be confirmed by analyzing the morphological status of the cells or by comparing the metabolite content of a non-quenched vs a quenched CF fraction. However, the fractions of extracellular pools varied across the different metabolites and some of them were really low, indicating that the presence of these metabolic intermediates in the culture medium cannot be explained only by cell lysis. Finally the fractions of extracellular metabolites obtained with our quenching method were in the same range of the ones obtained for *E. coli* without stainless steel beads [22], suggesting that leakage may not only be caused by the beads. According to our data, fewer metabolites were released at lower quenching temperature, highlighting that in *B. methanolicus*, leakage may not be primarily caused by cold shock. Further investigation is thus required to identify the factor(s) responsible for metabolite leakage in *B. methanolicus*. Overall this results pinpointed significant metabolites leakage with cell separation method which is consistent with previous data [21]. However, the average time between sample collection and extraction for the method with cell separation is about double that for the method without cell separation. We thus cannot exclude that this extra timing can cause extra cell stress partially explaining the high metabolite leakage observed.

#### Consistency of the measurements

The consistency of each metabolite measurement was evaluated under the elemental mass balance constraint (Eq. 1 in Sect. ‘‘Methods’’) which should be fulfilled if the quantification is correct. Except for ATP and citrate (Cit), the h index was below 9.49 (i.e. the limit value to accept no mismatches on quantification), indicating that quantification was consistent whatever the protocol used (Table 2). Except for citrate and ATP, metabolite levels were almost similar in the WB fraction than in the QC + WS fractions indicating proper sampling and quantification. In the case of citrate, the gross error measurement can be explained by external contamination which led to higher amount of citrate in the WS fraction (Fig. 2), most probably from the filter. In contrast, the amounts of ATP measured in the cell extract plus the washing solution (QC + WS) were lower than the amounts measured in the WB, indicating loss of ATP during sample treatment, which could not be corrected by the use of fully labelled ATP as internal standard (Fig. 2). In addition, ATP was apparently not converted into ADP or AMP, since no significant increase in those pools was observed. Overall, the consistency test showed that 16 out of 18 metabolites tested for displayed no quantification mismatches, highlighting the quality of the measurements.

#### Precision of the protocol without cell separation

**Fig. 3:**
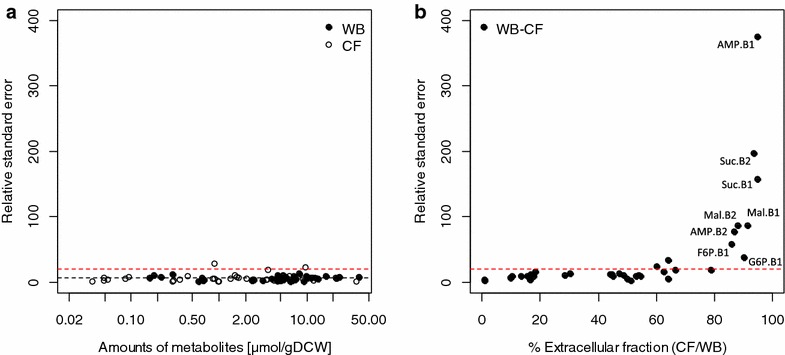
Precision of the metabolite quantifications by differential method in *B. methanolicus*. **a** Precision of metabolite measurements performed on WB and CF samples. *Black dashed line* represents the average of WB and CF relative standard error. **b** Precision of intracellular pool measurements obtained using the differential method (WB-CF) versus the extracellular fraction of each specific metabolite. *Red dashed line* represents the relative standard deviation threshold for acceptable precision (i.e. 20 %). Fumarate values were not plotted because its RSE were over 400. B1 and B2 state for biological replica 1 and 2. The raw data are given in Additional file 1

In previous sections, we demonstrated that when *B. methanolicus* cells are separated from the medium prior extraction, most of the metabolites are lost in the washing solution. Therefore the method without cell separation prior extraction is preferable for the measurement of its metabolome. However, the precision of the quantification using such approach is strongly affected by the concentration of metabolites outside the cells. The effect on measurement precision can be seen in Fig. 3. While separate measurements of WB and CF showed a relative standard error (RSE) below 20 % (Fig. 3a), the RSE of intracellular metabolite levels obtained by combining the two measurements was higher (Fig. 3b). This was particularly true for metabolites found in significant amounts in the culture filtrates (i.e. outside the cell) such as glucose-6-phosphate (G6P), AMP and TCA intermediates (Table 2). However, for metabolites with extracellular fractions below 50–60 %, RSE remained below 20 %.

### Test case: methanol derived metabolome analysis under different growth rates

In this study we optimized a procedure for metabolomics analysis of the thermophilic methylotroph *B. methanolicus*. This procedure is based on the quenching the whole broth with cold stainless-steel beads and subsequent extraction of the metabolites in a cold methanol/acetonitrile solution. To validate our protocol, we applied it on four *B. methanolicus* cultivations on methanol in chemostat at two different growth rates, 0.10 and 0.15 h^−1^.

#### Physiological parameters

The rates of methanol and oxygen consumption and the rates of biomass and carbon dioxide production were calculated along with methanol evaporation rates at steady state. The consistency of the experimental data was checked using standard data reconciliation procedures, under the constraint that elemental conservation relations were satisfied [31, 32]. No proof of mismatch was found for calculated rates. Under pure aerobic methylotrophic growth, the only products produced by *B. methanolicus* were biomass and CO_2_ (Table 3). In these conditions, methanol was almost completely consumed by the cells and less than 3 % was evaporated. As expected, oxygen consumption was higher than CO_2_ production, leading to a RQ coefficient lower than 1, which is characteristic of reduced carbon sources like methanol [3]. Interestingly, biomass yield increased with an increase in the growth rate by reducing the production of CO_2_. In general, the resulting physiological parameters were in agreement with previously published data obtained in the same conditions (Table 3) [38].

**Table 3:** Physiological parameters of *B. methanolicus* MGA3 chemostat cultures growing at two different dilution rates

	D: 010 h^−1a^	D: 015 h^−1a^	From [38]^a^
	Value ± sd	Value ± sd	
Methanol [mmol/(gDCW*h)]	−7.48 ± 0.13	−8.59 ± 0.17	−15.5
Biomass [1/h]	0.100 ± 0.03	0.148 ± 0.001	0.25
CER [mmol/(gDCW*h)]	3.22 ± 0.2	2.60 ± 0.13	4.17
OUR [mmol/(gDCW*h)]	−7.05 ± 0.65	−6.76 ± 0.23	−10.82
MER [mmol/(gDCW*h)]	0.18 ± 0.07	0.08 ± 0.02	–
RQ [mol CO_2_/mol O_2_]	0.46 ± 0.01	0.38 ± 0.01	0.39
Yield [gDCW/gMeOH]	0.42 ± 0.02	0.53 ± 0.02	0.5

*sd* standard deviation; *CER* CO_2_ evolution rate; *OUR* O_2_ uptake rate; *MER* methanol evaporation rate; *RQ* respiratory coefficient

^a^Positive values mean production and negative values mean consumption

#### Intracellular metabolome

Comparisons of the intracellular metabolite pools of *B. methanolicus* grown at different growth rates showed no significant differences, except in the case of citric acid (Cit) and PEP, where intracellular metabolites were higher at lower growth rates (Table 4). This is probably due to the low difference between the two tested conditions. However, similar features of methanol metabolism were observed as previously observed [39, 40]. Fructose-1,6-bisphosphate (FBP) was the most abundant metabolite irrespective of the culture conditions and in the same order of magnitude as in a previous metabolomics dataset obtained for this specific methylotroph grown in batch on methanol at 50 °C (Table 4, [39]). These data are consistent with the low affinity for FBP of plasmidic fructose 1,6-bisphosphatase isoenzymes upregulated upon methanol growth [6, 8, 40]. Despite the low precision of hexose-6-phosphate (H6P) values due to the significant quantities outside the cell (Table 2), their sum (G6P + F6P + M6P) was quiet close to amount of H6P observed in the previous dataset. Except for pentose-5-phosphate pool (R5P), pool sizes of the intermediates of the pentose phosphate (PP) pathway were in the same range but slightly higher than the ones observed previously [39]. This is consistent with the fact that former data were obtained by fast filtration, thus lower levels due to leakage may be expected. However, this difference may be also due to the difference in culture conditions rather than the method itself. The previous study used batch culture while the presented data were obtained in a methanol limited condition. TCA cycle metabolites were found in smaller amounts in *B. methanolicus* than in pure glycolytic metabolism, in agreement with low TCA activity in methylotrophic bacteria [41–43]. Finally, the calculated adenylate energy charges (AECs) were slightly below but not significantly different from 0.80, a value considered as characteristic of energetically healthy cells [44]. Overall our procedure resulted in reliable and reproducible data that are not significantly different from those published previously, supporting the validity of our protocol.

**Table 4:** Levels of intracellular metabolites and adenylate energy charge (AEC) of *B. methanolicus MGA* grown on methanol at D: 0.15 h^−1^ and 0.10 h^−1^

Intracellular metabolite pools [µmol/gDCW ± se]					
PEP	D: 0.10 h^−1^	D: 0.10 h^−1^	D: 0.15 h^−1^	D: 0.15 h^−1^	From [39]
	0.85 ± 0.05	1.12 ± 0.11	0.55 ± 0.05	0.48 ± 0.02	
P-Ser	0.18 ± 0.01	0.25 ± 0.03	0.26 ± 0.00	0.28 ± 0.04	
23PG	2.40 ± 0.11	2.98 ± 0.29	2.05 ± 0.15	2.21 ± 0.08	
R5P	2.18 ± 0.36	3.59 ± 0.38	3.29 ± 0.16	2.94 ± 0.53	0.49 ± 0.09
Shi3P	0.07 ± 0.01	0.08 ± 0.01	0.09 ± 0.01	0.10 ± 0.01	
F1P	0.24 ± 0.06	0.37 ± 0.05	0.18 ± 0.02	0.15 ± 0.06	
G6P	0.97 ± 0.38	1.73 ± 0.32	1.04 ± 0.53	1.38 ± 0.52	2.86 ± 0.25^a^
F6P	1.58 ± 0.92	3.54 ± 1.18	1.33 ± 0.48	1.70 ± 0.39	
M6P	0.22 ± 0.04	0.34 ± 0.04	0.40 ± 0.02	0.45 ± 0.07	
6PG	2.25 ± 0.25	3.80 ± 0.63	1.90 ± 0.08	1.71 ± 0.12	1.62 ± 0.13
S7P	4.45 ± 0.11	5.62 ± 0.70	4.04 ± 0.19	3.45 ± 0.15	3.55 ± 0.31
FBP	14.61 ± 1.30	19.35 ± 1.21	14.65 ± 0.28	13.80 ± 1.89	16.28 ± 1.30
AMP	0.64 ± 2.42	1.75 ± 1.34	1.02 ± 0.19	1.35 ± 0.46	
ADP	5.47 ± 0.53	6.31 ± 0.58	5.04 ± 0.17	5.44 ± 0.57	
ATP	6.79 ± 0.13	7.33 ± 0.32	7.33 ± 0.39	6.33 ± 0.45	
Fum	0.02 ± 0.51	0.04 ± 0.30	0.20 ± 0.09	0.89 ± 0.21	
Mal	0.41 ± 0.35	0.48 ± 0.42	0.47 ± 0.09	0.65 ± 0.02	
Cit	4.10 ± 0.39	3.70 ± 0.52	2.87 ± 0.08	2.31 ± 0.35	
AEC	0.74 ± 0.26	0.68 ± 0.15	0.74 ± 0.07	0.69 ± 0.13	

Each chemostat culture was considered separately. The metabolite abbreviations and the raw data are given in Additional file 1

^a^This is the concentration of the pool of hexose-6-phosphate

## Conclusion

We demonstrated that, like in many prokaryotic cells, significant leakage of metabolites occurs in *B. methanolicus*, thus might hampering the use of protocols which include cell separation for metabolomics. For proper quantitative metabolomics studies in this methylotrophic organism, total broth quenching with correction for the metabolites present in the extracellular medium proved to be a good alternative and might be an improvement on fast filtration based approach. The use of this protocol for steady-state chemostat cultures yielded accurate, reliable and valuable datasets to assess the use of methanol by B*. methanolicus* at 50 °C. These results are the first step toward the better system-level understanding of methanol-derived metabolism in this thermophilic gram-positive bacterium. We focused in this study metabolites closely related with the central methanol metabolism; however more quenching and extracting methods must be tested to expand the number of metabolites that could be measured. Anyway, by providing access to the metabolome of *B. methanolicus* in a quantitative manner, this work paves the way to rationally shape its metabolism for the efficient use of methanol as raw material in biotechnology.

## Additional file

10.1186/s12934-016-0483-x: List of metabolites with their abbreviations and InchI Code and InchI Key, raw data (i.e. analyte peak area and the corresponding concentration) and calibration coefficients.

## Abbreviations

S7P: sedoheptulose 7-phosphate
PEP: phosphoenolpyruvate
23 PG: 2 + 3 phosphoglycerate
R5P: ribose 5-phosphate + ribulose 5-phosphate + xylulose 5-phosphate
G6P: glucose 6-phosphate
F1P: fructose 1-phosphate
F6P: fructose 6-phosphate
M6P: mannose 6-phosphate
6PG: 6-phosphogluconate
FBP: fructose-16-bisphosphate
AMP: adenosine 5′-monophosphate
ADP: adenosine diphosphate
ATP: adenosine triphosphate
P-serine: *O*-l-phosphoserine
Shi3P: shikimate 3P
Fum: fumarate
Mal: malate
Cit: citric acid
RuMP: ribulose monophosphate
